# Higher Medical Education

**Published:** 1888-08

**Authors:** 


### EDITORIAL.

### HIGHER MEDICAL EDUCATION.

The season of the year has come again when the various medical colleges are sending forth their annual announcements, giving a statement of the advantages and the curriculum of each, and their requirements for obtaining the degree of Doctor of Medicine.

It is a gratifying assurance of advancement, for which a large part of the profession has been pleading for years, to see the gradual increase in the number of the older and stronger medical colleges that are requiring evidences of proper mental training for admission to them; that are lengthening the period of study requisite for a degree in medicine; that are increasing their facilities for instruction, and making their examinations more rigid.

The announcements which have come to the *Journal and Examiner* during this season show that some of the more prominent schools to which the profession has been looking to see evidences of their intention to keep pace with the advances that others are making, now show that those institutions are declaring their intentions to do so, immediately or in the near future, and for this they are to be commended.

In the annual address delivered at the last meeting of the Massachusetts State Medical Society, Dr. B. J. Jeffries sought to show how the medical profession has lost some of the position which it held in past times. Extracts from that address appear in another part of this number of the *Journal and Examiner.* Whilst conceding that it is true that never have the opportunities and the facilities for gaining a liberal medical education been so great as at the present time, he believes that the advances made in late years in all walks of life have rendered it incumbent on the physician to make the most of those increased facilities, since more is expected of him.

Without the broad and liberal culture which those facilities can bring, the medical profession must expect to yield some of the position it has held in the learned professions.

That too many had gained access to its ranks who either had not enjoyed those advantages, or who had neglected to avail themselves of them and properly profit by by them, he believed to be one of the causes which have been tending to lower the standing of the whole medical profession in the estimation of the general public. His suggestions for the remedy of this condition, and restoration of the profession to its former proud position, are in accord with the ideas of many others who have been striving to keep our profession in the front in the great advance that this century is witnessing in every phase of life.

The first requisite for this must, of course, be the natural capacity for such work, and the proper discipline of those faculties prior to beginning the study of medicine; then adequate facilities for the right kind of instruction, such as our best colleges now possess, and the prosecution of study, theoretical and clinical, under such circumstances for a sufficient number of years to enable the mind to comprehend the undertaking.

Whilst in an extensive country like ours, with its varying demands upon its physicians, no uniform standard is practicable, yet to maintain the position which all wish to see it hold, the percentage of liberally educated men in it must be large to secure that dominating influence which alone can keep the standard high.

The *Journal and Examiner* would offer no discouragement to any of those colleges that are earnestly striving to uphold a high standard, and to well qualify young men for their responsible calling, but it would especially commend the efforts of the colleges, whose age and standing make them conspicuous, that are giving evidence of being in sympathy with the demands of the times for the highest standard of medical attainment that is practicable, and to express the desire that the profession at large, whose support is essential for their fruition, show appreciation of those efforts. At least temporary sacrifices attend such struggles for advancement, but by giving proper support to such as maintain the highest standard, others will be encouraged to do likewise.

Dr. Jeffries might have gone still further in accounting for the assumed lowerering of the standing of the medical profession, and stated that the medical colleges are not simply teaching bodies, but that they have the power to confer degrees, and that their degree of Doctor of Medicine, with the assurance given in the diploma granted, entitles the holder of it to practice medicine in most of the States and Territories without question. Should a time ever come when the function of a medical college should be exclusively that of teaching, and the licensing power be vested in an entirely distinct body, whose duty it would be to ascertain the qualifications of candidates for a license to practice medicine, a most unwelcome duty would be removed from the faculties of the colleges, who are now forced to reject many candidates for a degree who have been their own pupils. Whilst there are arguments, and forcible ones, on both sides of this question, it seems not improbable that making colleges simply teaching bodies would result in a higher average of medical attainment and thus bring into the medical profession a larger percentage of liberally educated men who would be able to command the confidence and the respect of the world at large and maintain the high standing of the medical profession, whatever might be that of the other professions to which Dr. Jeffries' address alluded.

